# Comparison of Ultrasound-Guided Versus Anatomical Landmark-Guided Thoracolumbar Retrolaminar Techniques in Canine Cadavers

**DOI:** 10.3390/ani13193045

**Published:** 2023-09-28

**Authors:** Julia Pentsou, Séamus Hoey, Michail Vagias, Bethany Guy, Vilhelmiina Huuskonen

**Affiliations:** 1 Department of Veterinary Anaesthesia and Analgesia, Royal (Dick) School of Veterinary Studies, University of Edinburgh, Edinburgh EH25 9RG, UK; 2 Equine Clinical Studies, Diagnostic Imaging and Anaesthesia, UCD School of Veterinary Medicine, University College Dublin, D04W6F6 Dublin, Ireland; seamus.hoey@ucd.ie (S.H.); vilhelmiina.huuskonen@ucd.ie (V.H.); 3 Department of Small Animal Surgery, Royal (Dick) School of Veterinary Studies, University of Edinburgh, Edinburgh EH25 9RG, UK; mvagias2@ed.ac.uk; 4 Department of Veterinary Medicine, Queen’s Veterinary School Hospital, University of Cambridge, Madingley Road, Cambridge CB3 0ES, UK; bg447@cam.ac.uk

**Keywords:** dogs, fascial plane block, retrolaminar block, paravertebral, thoracolumbar analgesia, ultrasound-guided

## Abstract

**Simple Summary:**

Ultrasound guidance enables direct visualisation of local anaesthetic spread during the performance of regional anaesthetic techniques, thus improving their accuracy and effectiveness while minimising their complications. In human patients receiving thoracic analgesia, a new regional anaesthetic technique, named retrolaminar block, has a lower complication risk when compared with the gold standard, thoracic paravertebral block. The aim of this study was to develop an ultrasound-guided thoracolumbar retrolaminar technique to provide thoracolumbar analgesia in dogs. Another aim was to compare the distribution of contrast solution between the ultrasound-guided and landmark-guided techniques in canine cadavers, using computed tomography. Ten canine cadavers were randomised to receive either an ultrasound-guided or a landmark-guided retrolaminar injection of 0.6 mL/kg of iodinated contrast solution in each hemithorax at the level of the twelfth thoracic vertebra. The injectate spread in a similar manner in the retrolaminar space and the space surrounding the intervertebral foramina, while minimal spread was noted in the paravertebral space. One of the landmark-guided injections failed to spread, thus highlighting the importance of ultrasound guidance in improving outcomes in regional anaesthetic techniques. Further studies are warranted to clarify the role of the ultrasound-guided retrolaminar technique in the management of thoracolumbar pain in dogs.

**Abstract:**

The retrolaminar block was developed in humans as an easier and safer alternative to the thoracic paravertebral block. This study aims to describe an ultrasound-guided thoracolumbar retrolaminar injection in canine cadavers and compare the injectate distribution between a landmark-guided and an ultrasound-guided thoracolumbar retrolaminar technique using computed tomography. Ten canine cadavers were randomised to receive two injections each of 0.6 mL/kg of iodinated contrast at the level of the twelfth thoracic vertebra (T12): a landmark-guided retrolaminar injection was performed on one hemithorax (group B, *n* = 10) and an ultrasound-guided on the other hemithorax (group U, *n* = 10). Groups were compared using the Mann–Whitney *U* test. The median (range) spread of the contrast in the paravertebral space was 0 (0–3) and 1 (0–5) vertebrae in groups B and U, respectively (*p* = 0.038). The median (range) extent of the spread surrounding the interverbal foramina was 4 (0–5) in group B and 4 (3–5) in group U. The median (range) spread along the retrolaminar space cranial and caudal to T12 was 3 (0–6) retrolaminar segments in group B and 3 (3–4) in group U. The potential of the ultrasound-guided retrolaminar injection to provide analgesia for dogs suffering from thoracolumbar pain should be further investigated.

## 1. Introduction

Over the past two decades, the benefits of regional anaesthesia have been well established in human patients [1]. Some of those benefits include, but are not limited to, reduction of acute and chronic pain, reduction of postoperative nausea and vomiting, reduction in hospitalisation period, and overall increased patient satisfaction [2]. Following in the footsteps of human anaesthesia, veterinary regional anaesthesia is continuously developing [3].

The key requirement for a successful regional anaesthetic intervention is based on the optimal deposition of the local anaesthetic around the target tissues whether they are nerve structures, plexi, or, more recently, fascial planes [4,5]. There are several techniques to localise peripheral nerves, but the ones most commonly used in veterinary practice are anatomical landmarks, electrical nerve stimulation, and most recently, ultrasound guidance [6,7].

The utilisation of topographical landmarks to assist anaesthesiologists during procedural care includes various regional nerve blocks, neuraxial techniques, and interventional pain procedures. These methods are based on a thorough understanding of anatomic relationships to effectively deliver local anaesthetics and avoid morbidity and mortality [8]. Additionally, some regional and neuraxial techniques such as the epidural are based on the sensation of the passage of the needle through different tissue planes, something that is intuitively subjective [9]. The limitations of utilising anatomical landmarks lie in the inherent failure of the technique, due to anatomical variation, potential iatrogenic nerve damage, and unreliable spread of the local anaesthetic [10].

Most recently, ultrasonography has emerged as a method to locate peripheral nerves. Ultrasound-guided regional anaesthesia is an exciting and relatively new technique that is currently rapidly developing and allows for the visualisation of the target nerves or structures, the approaching needles, and the deposition of local anaesthetic in close proximity to the nerve in real-time [10]. Thus, the incorporation of this modality can allow for more accurate nerve location and improving block success rates irrespective of individual anatomic variations. It can also help to avoid critical anatomical structures such as blood vessels, hollow organs or pleura, and reduce the amount of local anaesthetic needed, resulting in safer drug administration [11]. Conversely, the use of ultrasonography in regional anaesthesia requires a good understanding and knowledge of relevant anatomy and, most importantly, accurate interpretation of the two-dimensional image provided by the ultrasound of a three-dimensional structure. Additionally, manual dexterity of the operator is necessary during the movement of the needle towards the target nerve, which makes the technique notoriously operator-dependent [9].

In an attempt to develop a simpler and safer landmark-guided alternative approach to the thoracic paravertebral block in humans, Pfeiffer and colleagues (2006) described what is known today as the retrolaminar fascial plane block [12,13]. During the retrolaminar injection, the local anaesthetic is deposited directly on the dorsal aspect of the target vertebral lamina, between the lamina and the overlying epaxial muscles, into the retrolaminar fascial plane, without the needle ever entering into the thoracic paravertebral space [14]. A previous study has evaluated the distribution pattern of two different injectate volumes following a thoracolumbar anatomical landmark-guided retrolaminar injection in greyhound cadavers [15]. Recently, the retrolaminar technique has also been performed as part of multimodal analgesia management in seven clinical canine patients undergoing thoracolumbar spinal surgery [16].

The objectives of this study were to describe an ultrasound-guided approach to performing a thoracolumbar retrolaminar injection and demonstrate the feasibility and potential complications of the technique in canine cadavers. A further objective was to compare the distribution of the injectate between the ultrasound-guided and a landmark-guided technique using computed tomography.

The hypothesis was that identification of the ultrasonographical landmarks to perform the retrolaminar technique would be easy, and that ultrasound guidance would be useful in optimising needle position and improving accuracy.

## 2. Materials and Methods

### 2.1. Animals and Study Design

This study was granted an exemption from full ethical review by the Animal Research Ethics Committee of University College Dublin (AREC-E-20-28-Huuskonen).

The cadavers of ten adult greyhound dogs, that had been euthanised for reasons unrelated to the present study, frozen, and subsequently thawed at ambient temperature for 72–96 h, were used. Cadavers with spinal abnormalities that could have hindered the identification of the anatomical or ultrasonographic target landmarks were excluded from the study. All cadavers were randomly assigned to receive an anatomical landmark-guided retrolaminar injection in one hemithorax (group B, *n* = 10) and an ultrasound-guided in the other (group U, *n* = 10) using a random sequence generator (www.randomizer.org, accessed on 26 August 2023). A total volume of 0.6 mL/kg iodinated contrast (ioversol) (Optiray^®^, 300 mg I/mL, Guerbet, Villepinte, France) was injected per site. In all cadavers, the ultrasound-guided injection was performed first, immediately followed by the anatomical landmark-guided retrolaminar injection. The distribution of the injectate was evaluated using computed tomography (CT) immediately after the injections were performed.

### 2.2. Identification of the Thoracolumbar Retrolaminar Space

**Figure 1:**
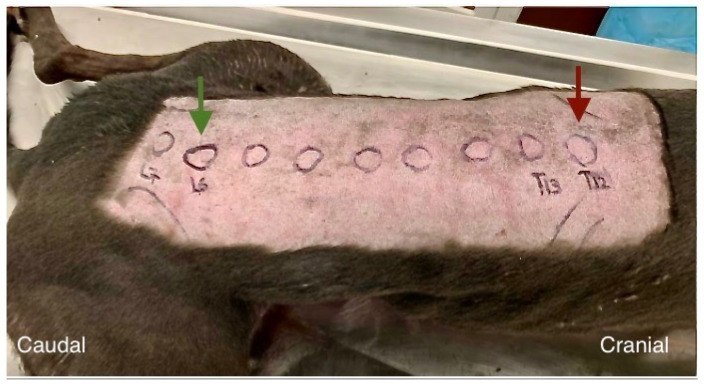
Cadaver preparation for the injections. The cadavers (cadaver 1 pictured) were placed in sternal recumbency. After clipping the hair, the sixth lumbar vertebral spinous process was identified by palpation (green arrow) and then the palpation continued cranially until the twelfth thoracic vertebral spinous process was found (red arrow).

All cadavers were weighed and placed in sternal recumbency for the injections. The hair was clipped from the mid-thoracic region to the lumbosacral region. The wings of the ileum and the spinous process of the sixth lumbar vertebra were located by palpation, and the palpation extended cranially to the lumbar and thoracic spinous processes, up to the level of the twelfth thoracic vertebral spinous process (T12). The identification of the landmarks was confirmed by two investigators blinded to each other (JP and MV). The spinous processes were then marked with a surgical skin marker (Figure 1). The fascial plane was defined between the bony vertebral lamina and the muscles above it as the retrolaminar space.

#### 2.2.1. Anatomical Landmark-Guided Retrolaminar Injections

With the cadaver in sternal recumbency and the T12 already identified, an 18-gauge, 3.5-inch spinal needle (Quincke disposable spinal needle, BD, Eysins, Switzerland) was introduced next to the spinous process through the epaxial muscles while the needle was maintained in a strict parasagittal plane, approximately 1–1.5 cm lateral to the spinous process. The needle orientation was caudoventral, at a 45° angle to the skin, aiming towards the retrolaminar space, and it was advanced until contact with the T12 vertebral lamina was achieved. The advancement of the needle was then stopped, a 20 mL syringe (Omnifix, Braun Medical, Melsungen, Germany) containing the iodinated contrast was then connected to the needle, and, following a negative air and blood aspiration, the retrolaminar injections were performed by the same investigator (JP). To detect an accidental pleural, epidural, or paravertebral puncture, the loss of resistance technique was used during the injection, in which the injection would be stopped and the needle repositioned if an increased resistance was detected during injection. The depth at which the needle was inserted was approximately 1.5–2 cm, depending on the body condition of the cadaver.

#### 2.2.2. Ultrasound-Guided Retrolaminar Injections

**Figure 2:**
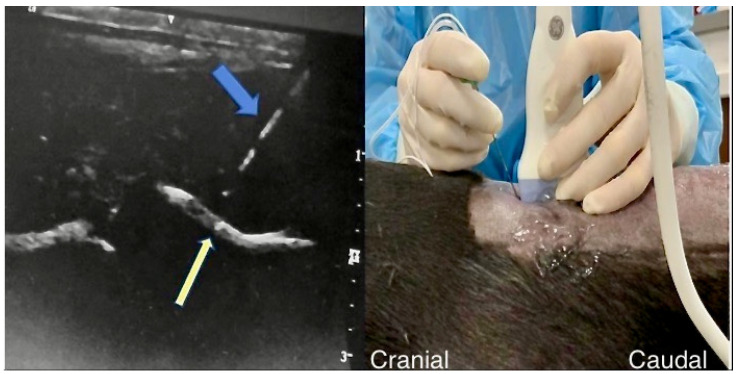
Ultrasonographic appearance and positioning of the ultrasound transducer during a T12 retrolaminar injection. On the (**left**), the yellow arrow depicts the T12 vertebral lamina, and the blue arrow shows the needle as it approaches the dorsal aspect of the lamina. On the (**right**) is the sagittal position of the ultrasound transducer on a dog cadaver in sternal recumbency as the needle is advanced through the thoracic epaxial muscles to approach the T12 lamina.

All ultrasonographic examinations were conducted using the same ultrasound machine (GE LOGIC e R7, GE Healthcare, Chicago, IL, USA) with a linear ultrasound transducer (12L-RS, 5–13 MHz, GE Healthcare, Chicago, IL, USA). The ultrasound probe was positioned in sagittal orientation approximately 1–1.5 cm lateral to the dorsal midline. The transducer was moved in a medial-to-lateral direction in order to identify the hyperechoic spinous vertebral processes, the vertebral laminae, the transverse vertebral processes, and the thoracic pleura. An in-plane technique was used and a 21-gauge, 100 mm insulated needle (Stimuplex Ultra 360, B Braun Medical Ltd., Dublin, Ireland) was inserted in a cranial-to-caudal direction, through the epaxial muscles, in a 45° angle to the skin, with the goal of having the tip of the needle in direct contact with the dorsal aspect of the T12 vertebral lamina (Figure 2). Once the desired position was achieved, a 20 mL syringe (Omnifix, Braun Medical, Melsungen, Germany) containing the iodinated contrast was connected to the needle and the injections were performed as described above. All injections were performed by the same investigator (VH).

### 2.3. Computed Tomography

Following the performance of all retrolaminar injections, the cadavers were moved onto the CT scanner table. A CT examination of the thoracolumbar area was performed using a 16-slice CT scanner (SOMOTO Scope: version syngo CT VC40, Siemens, Munich, Germany) with the cadavers positioned in sternal recumbency. The acquisition parameters used to obtain the scans were: 0.75 mm slice thickness at 220 mAs and 130 KVp. Computed tomographic images were then viewed using an image analysis software (OsiriX, Version 6.0, Pixmeo, Bernex, Switzerland). One ECVDI and ACVR board-certified veterinary radiologist (SH) and a veterinary radiology resident (BG) evaluated the CT images by consensus.

In each injection, the features assessed using the CT images were the following: confirmation of injection at the T12 retrolaminar space (yes/no), contrast location in paravertebral space, extent of contrast surrounding ipsilateral intervertebral foramina, extent of distribution of contrast in ipsilateral retrolaminar space, contrast distribution pattern (linear, intercostal, or both), epidural contrast migration (yes/no), extent of epidural contrast migration, presence of contrast in pleural space (yes/no), presence of contrast in retroperitoneal space (yes/no), presence of IV contrast (yes/no), presence of pneumothorax (yes/no).

To confirm the correct location of the initial injection site, the primary injection site was determined as the region with the highest Hounsfield units (HU) [17]. The HU is a quantitative measure method of assessing radiodensity utilised to assist interpretation of CT images [18]. Moreover, in terms of linear contrast distribution pattern, a craniocaudal spread of the injectate along the paraspinous muscles was considered, and as an intercostal distribution pattern, a more lateral spread towards the intercostal muscles was used.

### 2.4. Statistical Analysis

Statistical analysis was performed using IBM SPSS 27 (IBM Statistics; Endicott, NY, USA) for macOS. The data were assessed for normality using the Shapiro–Wilk test. Each hemithoracic retrolaminar injection was considered an independent variable and analysed accordingly. The extent of staining of paravertebral spaces, the intervertebral foramina, and the retrolaminar spaces, and the presence of intravascular contrast were compared between the two groups (group B versus group U) using the Mann–Whitney *U* test. Results are summarised as median (range). Categorical data are presented as frequencies. All statistical test results were interpreted using a 5% level of significance.

## 3. Results

### 3.1. Retrolaminar Injections

Ten cadavers were used in total, three of whom were female, and seven were male. Their median (range) weight was 30.75 (26.5–36) kg, with a median (range) body condition score of 4 (3–5) out of 9 [19]. The T12 spinous vertebral process was easy to identify by both investigators (JP and VH). The relevant sonoanatomy (thoracolumbar vertebral laminae and epaxial muscles) was also readily identified and the in-plane echogenic needle position was clearly visualised prior to all ultrasound-guided retrolaminar injections. When the retrolaminar injections were performed, all anatomical landmark-guided injections had a normal resistance during injection, while all ultrasound-guided retrolaminar injections presented increased resistance during injection. Both initial and intermittent aspirations were negative for air and blood during all injections in both techniques (100%). The loss of resistance test was negative for all injections. No further complications were noted during the procedure.

### 3.2. Computed Tomography

**Figure 3:**
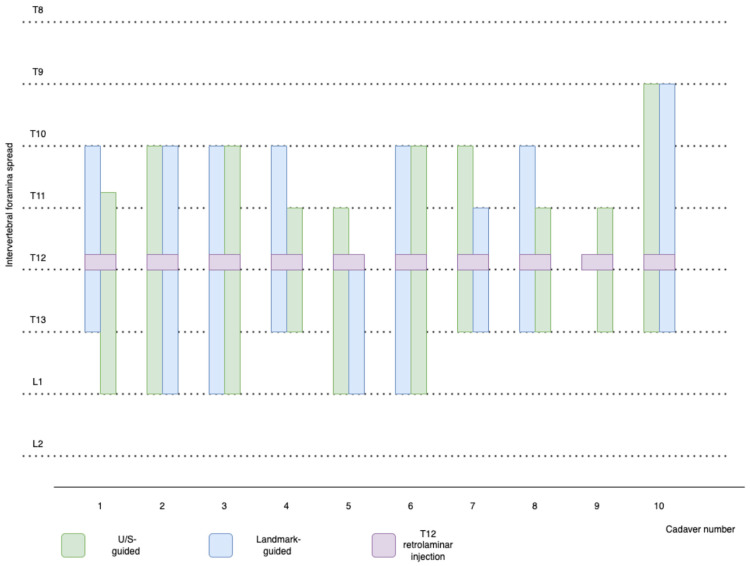
Description of the craniocaudal extent of the contrast to the thoracolumbar intervertebral foramina in ten canine cadavers, following either ultrasound-guided (green) or anatomical landmark-guided (blue) retrolaminar injections at the level of the twelfth thoracic vertebra (T12). The left side of each column indicates the left hemithorax and the right side indicates the right hemithorax of each cadaver. The purple colour represents the T12 retrolaminar injection. Note that the anatomical landmark-guided retrolaminar injection in cadaver 9 failed to spread sufficiently and surround any intervertebral foramina. U/S = ultrasound; T12 = twelfth thoracic vertebra.

The median (range) time between injection and the CT scan was 44 (31–56) min. The CT study confirmed that the injection site was always the intended one (T12 retrolaminar space) in all 20 injections. Contrast distribution was linear and intercostal for all cadavers except one that had only linear spread of the contrast. Concerning the presence of contrast in the underlying paravertebral space, the median (range) spread of contrast in the ultrasound-guided injections was 1 (0–5) paravertebral spaces, while for the anatomical landmark-guided injections, it was 0 (0–3), and this difference was statistically significant (*p* = 0.038). The median (range) spread of the contrast, cranial and caudal to T12, was 4 (3–5) intervertebral foramina for the ultrasound-guided group and 4 (0–5) for the anatomical landmark-guided group, with statistically significant difference between the two groups (*p* = 0.038) (Figure 3). The median (range) spread at the retrolaminar space and caudal to T12 was 3 (3–4) spaces for the ultrasound-guided group and 3 (0–6) spaces for the anatomical landmark-guided group, with no statistically significant difference between the two groups (*p* = 0.79). In one cadaver that received an anatomical landmark-guided retrolaminar injection, although contrast was identified in the target T12 lamina, it did not spread into the retrolaminar space or into the surrounding intervertebral foramina, rendering the injection unsuccessful (Table 1).

Epidural migration of the injectate was noted in two of the ten cadavers, with the contrast identified locally at the level of T12 in one (cadaver 2) and extending from T11 to T13 in the other (cadaver 10). No contrast was identified in the pleural space or in the retroperitoneal space in any of the ten cadavers.

In 18 out of the 20 retrolaminar injections, intravascular contrast was identified irrespective of the injection technique used, with no difference between the two groups (*p* = 0.93).

Pneumoperitoneum was identified in three cadavers and intravascular gas was noted in one cadaver. The volume of gas was significant.

No abnormality of the vertebral column that could have hindered the anatomical identification or the execution of the retrolaminar injection was identified in any of the cadavers.

## 4. Discussion

Following a search of the literature, this is the first study that describes an ultrasound-guided thoracolumbar retrolaminar technique in dogs, compares it with an anatomical landmark-guided approach, and evaluates the extent of the spread of 0.6 mL/kg of iodinated contrast in the intervertebral foramina, paravertebral space, and retrolaminar space after both techniques. The results of this study show that an ultrasound-guided retrolaminar injection in canine cadavers is feasible to perform, and that it is possible to use either anatomical landmarks or ultrasound guidance when approaching the retrolaminar space in dogs.

The findings of this study corroborate the hypothesis that ultrasound guidance would improve the accuracy of retrolaminar injections. Although there was no statistically significant difference between the two groups in the extent of the injectate spread in the retrolaminar space, the differences in the spread of the injectate around the intervertebral foramina and the paravertebral space were statistically significant between the two techniques. Furthermore, there was one unsuccessful injection in the anatomical landmark-guided group, where, although the contrast was identified in the T12 dorsal laminar space, it failed to spread into the retrolaminar fascial plane or around the intervertebral foramina. These findings highlight the advantage of ultrasound guidance as it allows real-time needle visualisation and injection in the target location confidently. In human studies, the addition of ultrasound did improve the retrolaminar technique at the T4 level [20], as identification of the needle and the surrounding anatomical structures decreased the chance of inadvertent pleural or epidural puncture and improved the accuracy of the delivery of the injectate to the target area [13]. The introduction of ultrasonography to regional anaesthesia has greatly supported the development of fascial plane blocks, where the target is a fascial plane rather than a particular nerve [14]. In this study, the use of in-plane ultrasound guidance allowed for the easy identification of the vertebral lamina in all ten canine cadavers, and, therefore, the technique could potentially be further investigated for its clinical efficacy. In humans, the retrolaminar block has been used successfully as a component of multimodal analgesia in rib fractures, thoracotomies, mastectomies, thoracoscopies, herniotomies, and, recently, spinal surgery [13,20,21,22,23].

The injectate did not consistently spread in the paravertebral space with either of the injection techniques used. In this study, only iodinated contrast was used for injections, making the viscosity of the injectate quite high, which could have potentially affected the injectate spread [24]. Previous studies have shown a clear association between paravertebral spread and the volume used for retrolaminar injections in human and porcine cadavers [25,26]. Potentially, the volumes used in this study were not adequate to allow for paravertebral spread. Nevertheless, human cadaveric studies have also identified variability in the paravertebral spread of retrolaminar injections [24,25]. This contradicts the original theory about the retrolaminar block exerting its analgesic effect as an alternative paravertebral technique [12,13,27] and further supports the theory that proposes that the spread of the local anaesthetic in the retrolaminar fascial plane, the plane between the dorsal laminar surface and the overlying epaxial muscle, is responsible for the clinically successful analgesic outcomes [28,29].

The epidural spread of the injectate was also minimal and very localised in this study. The epidural spread following a retrolaminar injection is an inconsistent finding in human studies [24,30]. Furthermore, in a previous study of the anatomical landmark-guided retrolaminar technique in canine cadavers, the epidural spread was present in seven out of eight cadavers [31]. However, the utilisation of ultrasound guidance should eliminate the possibility of accidental epidural injection. In addition, all retrolaminar injections in this study incorporated the loss of resistance test (LOR) to identify accidental epidural injection as an extra safety measure. The LOR technique has been recently utilised successfully in the performance of thoracic paravertebral injections in canine and feline cadavers [32]. The epidural spread noted could be a result of a passive spread of the injectate due to compromised tissue structure and high injection pressures.

High amounts of injectate were noted in the intravascular space irrespective of the injection technique used. In fact, in one of the cadavers, air was also identified intravascularly during the CT examination, which is believed to be a result of inevitable post mortem changes and loss of vascular integrity. In a previous cadaveric study of the anatomical landmark-guided retrolaminar technique, significant amounts of intravascular contrast were identified in vessels such as the azygos where direct intravascular injection would have been anatomically impossible [31]. The clinical significance of this finding remains unknown, but during the performance of both techniques, negative aspiration for air and blood was confirmed prior to injection; however, negative blood aspiration testing is not necessarily reliable when performed on cadavers, due to post mortem blood clotting. Moreover, in clinical patients, the ultrasound-guided retrolaminar technique would help with the identification of vessels in the overlying epaxial muscles, further adding to the safety of the technique and minimising the risk of intravascular injection of local anaesthetic agents.

There are several limitations to this study. First, the total number of cadaveric specimens (*n* = 10) was small, and a power calculation was not performed prior to the investigation. However, other studies in the past that have investigated regional anaesthetic techniques in canine cadavers have utilised a similar number of dogs [32,33], and, due to institutional ethical concerns, access to cadavers was limited. Moreover, the cadavers used were all greyhounds with similar body condition—a factor that can affect the generalisation of the findings to dogs of different breeds and anatomical conformation.

Furthermore, another limitation is the cadaveric nature of the study, which might have affected the spread of the injectate. Potentially, the use of fresh (nonfrozen) cadavers would have decreased the incidence of severely compromised tissue integrity that accompanies thawing frozen cadavers, but access to such resources was not available to us. Additionally, the synchronised movement of the chest cavity during normal ventilation is believed to affect the spread of the injectate in clinical retrolaminar injections in human patients [25], which could further limit the usefulness of cadaveric studies.

Finally, neither the pressure of injection nor the rate of injection was measured or standardised in either of the techniques. This could have also affected the injectate spread, especially since there was a subjective observation of increased resistance to injection during the ultrasound-guided technique. The increased resistance during injection was most likely a result of the increased viscosity of the injectate in combination with the smaller diameter needle used for the ultrasound-guided technique. In fact, the needle used for the ultrasound injections was 21-gauge, while the needle used for the landmark-guided injections was 18-gauge, and, according to Poiseuille’s law, resistance to flow is directly proportional to the fourth power of the radius and the viscosity of the fluid.

## 5. Conclusions

To conclude, ultrasound-guided thoracolumbar retrolaminar injections were technically simple to perform, and the relevant sonoanatomy was easy to visualise and identify in canine cadavers. All ultrasound-guided retrolaminar approaches were successful, whereas one anatomical landmark-guided injection was unsuccessful, thus supporting the hypothesis that ultrasound guidance provides additional safety and accuracy to regional anaesthetic techniques. The injectate distribution in the intended thoracolumbar retrolaminar space and intervertebral foramina area, where dorsal branches of the spinal nerves are located, should allow for analgesic provision in clinical scenarios. Further clinical studies should assist in a better understanding of the potential indications of this technique.

## Figures and Tables

**Table 1:** Comparison of the injectate spread along the intervertebral foramina, in the paravertebral space and in the retrolaminar space. The table also shows the spread in the epidural space of two cadavers. U-group = ultrasound-guided retrolaminar injection group; B-group = anatomical landmark-guided retrolaminar injection group; PV = paravertebral space; RL = retrolaminar space.

Cadaver	U-Group Foramina Spread	B-Group Foramina Spread	U-Group PV Spread	B-Group PV Spread	Epidural Spread	U-Group RL Spread	B-Group RL Spread
1	T11–L1	T10–T13	T12–T13	0	0	T11–T13	T10–T13
2	T10–L1	T10–L1	T10–L1	T12–L1	T12	T11–T13	T11–T13
3	T10–L1	T10–L1	T10	0	0	T10–T13	T12–L1
4	T11–T13	T10–T13	0	0	0	T11–T13	T11–T12
5	T11–L1	T12–L1	T10	0	0	T11–T13	T12–T13
6	T11–L1	T10–L1	0	0	0	T11–T13	T11–T13
7	T11–T13	T11–T13	T11	T11	0	T11–T13	T11–T13
8	T11–T13	T11–T13	0	0	0	T11–T13	T10–T13
9	T11–T13	0	T13–L1	0	0	T11–T13	0
10	T9–T13	T9–T13	T10–T12	0	T11–T13	T11–T13	T9–L1

## Data Availability

The data presented in this study are available in detail on request from the corresponding author.
